# Mindfulness and the aging brain: a proposed paradigm shift

**DOI:** 10.3389/fnagi.2014.00120

**Published:** 2014-06-24

**Authors:** Ruchika Shaurya Prakash, Angeline A. De Leon, Beth Patterson, Brittney L. Schirda, Alisha L. Janssen

**Affiliations:** Department of Psychology, The Ohio State UniversityColumbus, OH, USA

**Keywords:** aging, mindfulness, emotional control, cognitive control, neuroimaging

## Abstract

There has been a proliferation of cognitive training studies investigating the efficacy of various cognitive training paradigms as well as strategies for improving cognitive control in the elderly. While some have found support for the transfer of training, the majority of training studies show modest to no transfer effects. When transfer effects have been observed, the mechanisms contributing to enhanced functioning have been difficult to dissociate. In this review, we provide a theoretical rationale for the study of mindfulness in older adults as a particular type of training program designed to improve cognitive control by capitalizing on older adults’ acquired behavioral orientation toward higher socioemotional goals. Given the synergistic relationship between emotional and cognitive control processes, the paradoxical divergence in older adults’ functional trajectory in these respective domains, and the harmonious interplay of cognitive and emotional control embedded in the practice of mindfulness, we propose mindfulness training as an opportunistic approach to cultivating cognitive benefits in older adults. The study of mindfulness within aging, we argue, capitalizes on a fundamental finding of the socioemotional aging literature, namely the preferential change in motivational goals of older adults from ones involving future-oriented wants and desires to present-focused emotion regulation and gratification.

A central focus of the field of cognitive aging involves securing the quality of life of older adults. This requires, first, an examination and understanding of age-related changes in the fundamental processes of controlled regulatory behavior and secondly, the design and development of intervention programs to reduce such age-related decline in the controlled processes. The centerpiece of this controlled regulatory processing may be theoretically differentiated into the complementary processes of emotional control and cognitive control, which, interestingly show divergent functional trajectories with age. While cognitive control operations have been known to show a steady decline with increasing age (Park et al., 2002; Salthouse, 2010), emotional control, along with overall emotional satisfaction, is well-maintained and sometimes even enhanced in older adults (Carstensen et al., 2000; Charles, 2010; Hay and Diehl, 2011). Furthermore, while engaging in emotional regulation has costs for cognitive control task performance in young adults, older adults do not demonstrate reductions in cognitive control capacity following an emotion regulation instruction, thus suggesting an effortless engagement of such affective regulatory behaviors in older adults (Scheibe and Blanchard-Fields, 2009). In this review article, we provide a rationale for the study of mindfulness training, which, by fundamentally reducing the reactivity of the wandering mind (Bishop et al., 2004), we argue is capable of enhancing both emotional and cognitive control in the aging brain. Given the putative primacy of maintaining emotional well-being and control in older adults (Carstensen, 1993, 2006; Carstensen et al., 1999), mindfulness training, with its emphasis on present-focused attention and regulation of the habitual, reflexive tendencies of the mind, has the potential to enhance cognitive control operations in the elderly and the neural circuitry associated with it.

With the aging of the baby boomer generation (Gavrilov and Heuveline, 2003) and the increasing standard of efficacy to which older adults hold themselves, a critical understanding of the mechanistic organization of cognitive and emotional functioning and their potential flexibility across the developmental lifespan represents the core of current investigative efforts (Hertzog et al., 2009; Lustig et al., 2009; Park and Bischof, 2013). While the makers of brain training games argue the capability of such cognitive training programs to produce broad transfer effects, much of the systematic, scientific study of cognitive training interventions provide modest support for the increase in overall cognitive capabilities of older adults, with limited benefits to tasks of everyday functioning (Willis et al., 2006; Lustig et al., 2009). Recent reviews of the current state of cognitive training literature in the elderly underscores the importance of designing interventions that produce both far-reaching transfer effects and enable an understanding of the mechanisms behind such enhanced functioning (Lustig et al., 2009; Park and Bischof, 2013). While strategy training studies are specific and clearly articulate the mediating factors that might be responsible for the change in functioning, there has been a consistent failure to produce any far-reaching transfer effects in several of these studies (Dunlosky et al., 2003; Rebok et al., 2007). On the other hand, multi-modal intervention techniques, involving potential lifestyle changes (Fried et al., 2004; Noice et al., 2004; Park et al., 2014), show the most promising effects; however, the ability to clearly parse the mechanisms of action remains a limitation of such approaches. Thus, an ideal training program would be one which pragmatically focuses on older adults’ increased socioemotional drive for emotional well-being and satisfaction; demonstrates broad transfer effects to domains of cognitive functioning that directly influence everyday functioning; and facilitates the development of a mechanistic model that explains the role of mediating variables.

We hypothesize, based on the increasing scientific study of mindfulness training (Davidson, 2010; Williams, 2010; Vago and Silbersweig, 2012), that this training approach has the potential to enhance cognitive control and emotional control capabilities in older adults and modify the neural circuitry supporting this controlled regulatory processing. In this article, we provide an overview of the age-related alterations seen as a function of the developmental trajectory, outlining a rationale for the incorporation of both emotional and cognitive aspects of regulatory control in a training program targeting the elderly. We then review the growing literature on mindfulness training, raising critical themes emerging from the systematic investigation of the influence of mindfulness training on emotional and cognitive control. We end by suggesting directions for future research, specifically noting some of the methodological issues that require further attention in this growing field of research.

## Emotion-cognition paradox in aging

**Figure 1:**
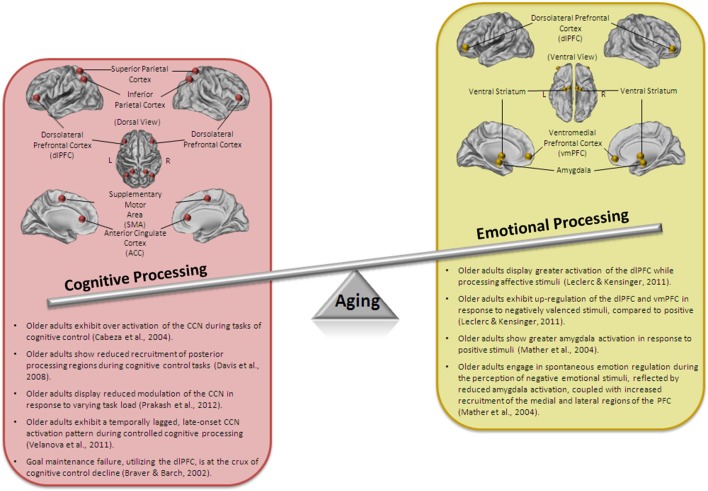
**Schematic representation of the aging paradox**. Older adults show a decline in cognitive processing, including impaired performance on tasks of cognitive control and alterations in the neural correlates of cognitive control. However, emotional processing, and at times, emotional regulation is well-maintained and even enhanced in older adults.

While sharing an integrated neuroarchitecture and operating using a similar dual-process system, when studied in the context of aging, cognitive and emotional control are remarkably divergent. A paradox that has long perplexed the aging research field is the emergence of an age-related enhancement in emotional efficacy in the midst of general diminishment of basic cognitive functioning (see Figure 1 for a summary of the key findings). It appears that, at the peak of human development, cognitive control capacity is at its greatest whereas emotion regulation ability is still developing, and that this trend is interestingly reversed with advanced age.

### Cognitive processing and aging

Emerging theories of healthy cognitive aging suggest that age-related variation in cognitive functioning is fundamentally linked to aging-based deficiencies in cognitive control (De Jong, 2001; Braver and West, 2008). There has been unequivocal evidence demonstrating that older adults, in comparison to young adults, consistently demonstrate poorer executive control capabilities, showing marked deficits in the ability to maintain task items online and/or quickly and accurately manipulate information (Park and Reuter-Lorenz, 2009; Goh et al., 2012). Cross-sectional and longitudinal research confirm that older adults experience difficulty performing tasks that require attending and responding to multiple sources of information, with age-related declines in executive processes occurring around 30 years of age and declining steadily across the lifespan (Park and Reuter-Lorenz, 2009).

One of the fundamental findings in this literature has been the inability of older adults to suppress task-irrelevant distractors (Hasher and Zacks, 1988; Gazzaley et al., 2005), suggesting that the impairment in controlled cognitive processing seems to arise from a disturbance in the top-down processing stream, caused by the intrusion of internal or external stimuli. Healthy aging is herein conceptualized as a problem in which the attentional filtering of older adults becomes increasingly diffuse so as to include not only relevant stimuli, but extraneous irrelevant stimuli as well. Older adults, thus show enhanced memory of distractor stimuli as well as attentional overprocessing of task-irrelevant information, which strongly correlates with diminished working memory performance (Gazzaley et al., 2005). Much of this, according to the goal maintenance account proposed by Braver et al. stems from an inability to maintain task-relevant, goal representations and thereby reduce interference from internal or external stimuli (Braver and Barch, 2002; Braver et al., 2005; Rush et al., 2006). In as far as goal maintenance represents a process of resistance to attentional interference, findings implicate that cognitive control decline in the elderly is indeed a problem of impaired top-down modulation, specifically in relation to the component associated with the controlled-attention mechanism of working memory (Gazzaley et al., 2005; Gazzaley, 2013).

Further, the goal maintenance account of cognitive control asserts the involvement of the lateral prefrontal cortices (lPFC) in representing and storing such contextual information (O’Reilly et al., 1999; Braver and Cohen, 2001). The successful exertion of cognitive control is thus, accomplished via the PFC’s exertion of top-down control on the posterior and subcortical brain regions involved in task-specific processing based on online goal representations (Miller and Cohen, 2001; Tamber-Rosenau et al., 2011). The lPFC is credited with not just the storage and maintenance of contextual representations in a highly accessible format, but also with the biasing of other, posterior cortical regions involved in task-specific processes, such as action selection and memory retrieval (Braver and Cohen, 2001; Braver et al., 2007). The concerted activity of the PFC and areas such as the anterior cingulate cortex (ACC), involved in interference detection; the posterior cortex, involved in the selective processing of sensory information and storage of domain-specific knowledge; and medial temporal lobe (MTL) structures, involved in associative binding and memory consolidation, enable the production of context-specific, goal-relevant behavior (Braver and Cohen, 2001).

The structural and functional deterioration of the PFC in older adults is indeed one of the most consistently reported findings in aging research (Cabeza, 2002; Raz et al., 2005; Ziegler et al., 2012). In older adults, the diminished ability for top-down modulation of irrelevant information is reflected by a general pattern of enhanced cortical activation in frontal regions (Cabeza, 2002; Grady et al., 2002; Colcombe et al., 2005; Prakash et al., 2009). On tasks of cognitive control, for example, older adults, relative to young, demonstrate cortical overactivation in areas of the task-positive, cognitive control network (CCN), comprised of dorsolateral prefrontal cortex (dlPFC), inferior parietal cortex, and supplementary motor area (Reuter-Lorenz et al., 2000; Cabeza, 2002; Cabeza et al., 2004). Moreover, older adults show a failure to modulate neural activity in these areas in response to increased task demands (Reuter-Lorenz and Mikels, 2006; Prakash et al., 2009), suggesting a general deficiency and inflexibility in the utilization of PFC-based cognitive control resources (Reuter-Lorenz and Park, 2010).

Accompanying the general over-recruitment of the PFC in aging, is the reduction in recruitment of the posterior processing regions (Davis et al., 2008), which could be indicative of a compensatory strategy of cortical recruitment. Alternatively, this could be reasonably reframed as being associated with an early disruption in goal maintenance, resulting in a failure to appropriately activate associative-sensory posterior regions in preparation for organized action. In addition to age-related shifts in the patterns of local brain activity and connectivity associated with cognitive control, there is also evidence for the utilization of a temporally-lagged, late-onset strategy in older adults during controlled cognitive processing, such that older adults show a failure to engage top-down attentional sets during early stages of a memory retrieval process, along with an increased recruitment of PFC regions during later stages (Velanova et al., 2007). Taken together, these neuroimaging studies provide collective support for the alteration in the spatial and temporal activity of the lateral prefrontal cortices, known to broadly serve cognitive control, and specifically the capacity to engage in goal-relevant task representations (Figure 1).

In fact, in a direct test of the goal-maintenance account, Paxton et al. (2008) examined the neural activity in the lPFC in older, relative to young adults during a modified version of the Continuous Performance Test (AX-CPT). Utilizing a combination of contextual, goal-relevant cues that demand maintenance of proactive control and ambiguous probes that require the re-activation of task-sets, thus eliciting reactive control, the task was designed to parse out the age-related variance in the recruitment of the lPFC during these two separate cognitive control strategies. Interestingly, while older adults showed sustained over-recruitment of the lPFC during the task, activity associated with goal maintenance during cue-related trials was diminished. These results were interpreted to suggest that aging is characterized by a shift in cognitive control strategies, such that older adults move from a more proactive cognitive control strategy which enables early selection of task-appropriate stimuli to a reactive control strategy which could either result in a temporally protracted response or a failure to inhibit task-irrelevant representations.

In further support of the goal-maintenance account, Gamboz et al. (2010) found age-related reductions primarily in the alerting component of the Attention Network Task (ANT; Fan et al., 2002). Usually employing some variant of the response compatibility paradigm, the ANT differentiates attention into three inter-related functions of alerting, orienting, and conflict resolution (Posner and Petersen, 1990). The alerting component taps into the ability to maintain an alert and stable task-state, presumably also invoking a representation of goals and task-related instructions. The evidence for an age-related decline in this component provides further support for the account that it is the maintenance of a vigilant, goal-relevant state that is compromised in older adults. Thus, a training program designed to increase moment-to-moment attention would be beneficial in enhancing cognitive control. Interestingly, in this study Gamboz et al. (2010) did not find evidence for an age-related reduction in the executive control component of the ANT contrary to many previously published studies reporting significant reductions in the ability to resolve response competition in older adults (Nielson et al., 2002; Colcombe et al., 2005).

One possible explanation for the discrepant findings in the executive control literature might have to do with the impact of perceptual load on the processing of goal relevant information (Lavie, 2005, 2010). According to the load theory of attention and cognitive control, the processing of irrelevant information or distractors is critically dependent on the perceptual load of the stimuli, such that higher perceptual load eliminates distractor interference, and low perceptual load can enhance the processing of irrelevant information, thereby increasing distractor interference (Lavie, 1995; Lavie and Cox, 1997). In fact, the extent to which tasks of low perceptual load result in distractor interference is further contingent on the frontal lobe capacities of individuals, such that older adults with compromised prefrontal cortex functioning show evidence of greater distractor interference than young adults at low perceptual loads (Maylor and Lavie, 1998). Thus, both perceptual load and executive load combined together can explain the mixed results of the cognitive aging literature and provide contextual evidence for a decline in executive control abilities in older adults.

To summarize, cognitive control capacities involving the meticulous and sustained recruitment of the lPFC are compromised in older adults, with deficits in goal maintenance lying at the crux of such cognitive control capacities. Training programs designed to specifically target enhancement of specific cognitive control abilities show limited transfer effects, presumably because of an inability to enhance moment-to-moment attentional control and simultaneously reduce distractions from internal and external disruptions. In addition, much of the training literature thus far is restricted by its failure to tap into the unique socioemotional motives which guide the behavior of older adults, therefore, such studies suffer from limited transfer effects. In order to determine whether a training program that enhances emotional well-being is circuitously able to influence the inter-related processes of cognitive control, the study of mindfulness in the elderly is crucial.

### Emotional processing and aging

The study of cognitive control in aging has been historically divorced from research focusing on the developmental trajectory of emotion processing and regulation in older adults. While unidimensional decline models of aging have been at the forefront of gerontological research, providing evidence for physical, physiological, and cognitive decline in the elderly, research in the field of emotional processing points to enhanced emotional well-being and satisfaction in older age (for a review see Charles and Carstensen, 2010 and see Figure 1 for a summary of the key findings). Despite having smaller social networks (Fung et al., 2001, 2008; Yeung et al., 2008), older adults report experiencing greater overall satisfaction and more positive than negative emotions in their social networks, relative to young adults (Carstensen, 1992; Newsom et al., 2005; Charles and Piazza, 2007).

Embedded within the construct of enhanced emotional well-being in older adults is their affinity towards positive rather than negative information, whether remembering real-life situations or stimuli in a laboratory-based task. When recalling the past, older adults tend to remember it more favorably than young adults (Kennedy et al., 2004). This age-related preference is observed even when remembering negative life events (Comblain et al., 2005), such that older adults give higher positive ratings of negative events, relative to the young cohort. Higher recall of positively-valenced laboratory stimuli has now been reported in several studies, and this shift from a predilection for negative information in young adulthood (Rozin and Royzman, 2001) to a heightened processing of positive stimuli, and reduced processing of negative information in older adulthood has been referred to as the “positivity effect” (Mather and Carstensen, 2003; Mikels et al., 2005). The positivity effect has been observed across a wide-range of laboratory-based tasks using a diverse array of emotional stimuli, and has been used to substantiate the hypothesis that aging is characterized by a greater preference for materials and attributes that are more positive than negative (e.g., Charles et al., 2003; Leigland et al., 2004; Mikels et al., 2005; Piguet et al., 2008; Shamaskin et al., 2010).

While a few theoretical accounts have been suggested for these findings, the socioemotional selectivity theory proposed by Carstensen et al. remains the dominant lens of interpretation (Carstensen, 1993, 2006; Carstensen et al., 1999; Reed and Carstensen, 2012). According to this theoretical model, a key developmental change that accounts for older adults’ acquired preference for positive over negative information is the change in goal orientation and motivation induced by the perceived narrowing of one’s scope of time as the temporal unfolding of life proceeds. With aging comes the inevitable realization of a limited time left in life, and thus, goals of emotional satisfaction and well-being take precedence over the future-oriented goals of achievement and self-expansion which most of young adulthood is geared toward. This shift in priorities, thus, leads to an increased focus on those attributes of life events and stimuli that would likely serve the higher-order, chronically-activated goals associated with ensuring well-being (Knight et al., 2007). Driven by these motivations and priorities, older adults, thus, selectively attend to positive stimuli and divert their attention away from negative information (Isaacowitz et al., 2006); are better able to positively appraise and dwell less on negative information than young adults (Charles and Carstensen, 2008); and engage in behaviors and emotion regulation strategies that support positive affect (Lefkowitz and Fingerman, 2003; Story et al., 2007; Löckenhoff and Carstensen, 2008).

To the extent that such selective preference is governed by adaptive higher-order goals related to emotional well-being, much of this age-related predilection appears to be under the influence of controlled regulatory processing. It may indeed be the case that cognitive control is a necessary pre-requisite for the successful implementation of the positivity effect (Mather and Carstensen, 2005; Kryla-Lighthall and Mather, 2009). In fact, research provides support for the positivity effect being a by-product of conscious, top-down regulatory processing, rather than being driven by more automatic, bottom-up processing. Individual differences in levels of cognitive capacity in older adults have been found to be associated with the tendency to favor positive pictures during memory recall (Mather and Knight, 2005). And interestingly, the preference for positive over negative pictures declines, once cognitive resources are directed to another task (Mather and Knight, 2005). More evidence for the delayed, conscious processing of positive over negative information comes from event-related potential studies that examine the temporal response to positive and neutral stimuli in older, relative to young adults (Williams et al., 2006; Isaacowitz et al., 2009). These studies suggest that the positivity effect is not present during rapidly presented emotionally salient stimuli, and when the effect is observed, it emerges much later after stimulus presentation, rather than directly following the onset of attention towards the positive stimuli.

The notion that the ability to successfully reappraise negative events is dependent on conscious cognitive control is further substantiated by the neuroimaging literature which consistently provides evidence for the enhanced engagement of the top-down neural circuitry of the prefrontal cortices in older adults during processing and reappraisal of affective stimuli (Urry et al., 2006; Williams et al., 2006; Leclerc and Kensinger, 2011). It is well known now that the neural circuitry of emotional processing involves key regions of the frontal cortex, specifically the ventromedial prefrontal cortex (vmPFC) and the dlPFC, as well as subcortical structures such as the amygdala and other association cortices (Phillips et al., 2003; Ochsner and Gross, 2008). The established structural and functional connections between this frontal-based, top-down network and subcortical bottom-up processing system represent the integrated neural infrastructure through which emotional responses are generated, coordinated, and modulated based on the allocation of cognitive resources (Ochsner et al., 2002, 2004). A failure in either system, resulting in either an amplification of the emotional cue’s saliency, via the activity of the amygdala or a failure to appropriately dampen emotional reactivity, through decreased top-down prefrontal activation may be sufficient to impede the successful deployment of emotional control (Rosenkranz et al., 2003; Goldin et al., 2008).

Research studies examining age-related differences in these neural regions supporting emotional processing now provide evidence for a three-way interaction between age, valence, and neural region (Gutchess et al., 2007; Ritchey et al., 2011; for a review, see Samanez-Larkin and Carstensen, 2011). While older adults demonstrate an up-regulation of the dlPFC and the vmPFC during negative, compared to positive pictures (Leclerc and Kensinger, 2011), they show an up-regulation in the amygdala during positive, relative to negative stimuli (Mather et al., 2004). Specifically, accumulating evidence suggests that even in the absence of instructed emotional regulation, older adults engage in spontaneous emotional regulation, reflected by reduced amygdala activation, coupled with increased recruitment of the frontal cortex, particularly in the medial and lateral areas of the prefrontal cortex, during the perception of negative emotional stimuli (Mather et al., 2004). However, when participants are instructed to engage in explicit emotional regulation, the findings are more mixed and seem to vary according to the strategies engaged by the task instructions. That is, older adults are more successful at regulating aversive affective stimuli when asked to engage in positive reappraisal and attentional deployment (Shiota and Levenson, 2009), rather than detached reappraisal (Winecoff et al., 2011). Further, when controlling for attentional deployment effects, older adults are less successful at using cognitive control to reappraise the negative stimuli than young adults (Opitz et al., 2012), despite a relatively greater frequency of engaging in cognitive reappraisal strategies (John and Gross, 2004).

Collectively, older adults do pay more attention to positive than negative stimuli and use certain selective emotional regulation strategies more effectively than others (Urry and Gross, 2010). While some of these age-related findings can be attributed to an increased reliance on structures of the prefrontal cortex that decline less structurally, such as the vmPFC (Raz, 2000; Fjell et al., 2009) and the ACC, the increased top-down neural recruitment of the dlPFC, despite its deteriorating capacity to maintain positive affect and decrease the influence of negative attributes, is consistent with the increased emphasis on emotional well-being in older adults (Mather, 2012). Another recent finding by Brassen et al. (2012) reporting reduced regret in emotionally healthy older adults following conditions of a decision task that elicited regret, suggests that one possible mechanism through which older adults might enhance emotional regulation is through the up- and down-regulation of positive affect (Suri and Gross, 2012). A training program designed to increase controlled regulatory processing in the elderly would, thus, need to tap into older adults’ higher-ordinate goals concerning emotional well-being and increased life-satisfaction. In the next section of this review article, we discuss the various facets of mindfulness training, with a special emphasis on the role of such training in enhancing emotional control and increasing overall life satisfaction.

## Mindfulness training and controlled regulatory processing

Interest in the construct of mindfulness has been gaining increasing momentum in the scientific world. Stemming from an interest in health and overall well-being, the study of mindfulness goes beyond the treatment of symptoms of psychopathology, which has historically dominated clinical science research. Individual differences in dispositional mindfulness or the cultivation of mindfulness skills through formal practices are now being systematically investigated to better understand the impact of this construct on individuals’ emotional and cognitive well-being (Brown and Ryan, 2003; de Vibe et al., 2012), physical health (Kabat-Zinn, 1982; Kabat-Zinn et al., 1985; Ludwig and Kabat-Zinn, 2008), immunological functioning (Davidson et al., 2003; Creswell et al., 2012), and overall quality of life (Nyklícek and Kuijpers, 2008; Krygier et al., 2013).

Embedded in the Buddhist tradition, the term “mindfulness” is one of the elements of the noble eightfold path articulated in the teachings of the Buddha (Bodhi, 2011). The complexities of this construct, as it was originally intended in the teachings of the Buddha have, until today, intrigued and perplexed Buddhist scholars. Therefore, much of the literature reviewed and presented here, stems from the contemporary definition of mindfulness that was first articulated by Jon Kabat-Zinn (1990, 2003).

Mindfulness, as defined in the contemporary sciences, is the practice of purposefully directing attention and in a non-judgmental way observing the unfolding of each moment as it takes place (Kabat-Zinn, 1994). The key facets of this concept involve a conscious choice to engage in the present-moment in its absolute entirety; bring an element of one-focused concentrative attention to the object(s) of the present-moment; and take the perspective of an impartial witness to the constantly changing cascade of thoughts, sensations, feelings, and other events. Importantly, it is critical that while performing all such activities, one does so by using the attitudinal principles of non-judgment, trust, patience, acceptance, non-striving, letting-go, and beginner’s mind (Kabat-Zinn, 1990).

While a number of meditation practices support the cultivation of the principles of mindfulness, focused attention and open monitoring are considered to be at the heart of many traditional 8 week mindfulness-based stress reduction (MBSR) programs (Lutz et al., 2007; Holzel et al., 2011). Utilizing the ever-present breath as an anchor, the practice of focused attention cultivates the development of sustained attention on the in- and out-flow of the breath. In the process of doing so, the goal of breath awareness practice is to stabilize the habitual, reflexive patterns of the wandering mind. These practices are hypothesized to enhance attentional control, and by learning to observe the natural process of the breath and extending this exercise to an entire array of experiences through the practice of open monitoring, the technique of mindful observation has been shown to enhance emotional regulation (Arch and Craske, 2006; Chambers et al., 2009; Farb et al., 2010). In fact, many recent models of mindfulness training conceptualize increased attentional control and emotional regulation as key mechanisms amongst others that support the cultivation of mindfulness (see Vago and Silbersweig, 2012 for an excellent review and theoretical conceptualization of the study of mindfulness).

**Figure 2:**
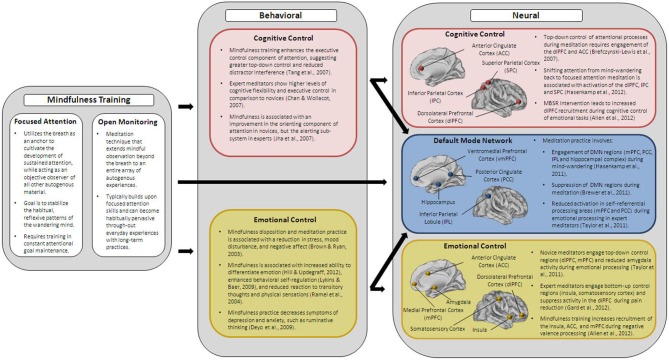
**Summarizes the key behavioral and neural findings of the effects of mindfulness training on cognitive and emotional control**.

Contextualizing the limited efficacy of current training paradigms in improving cognitive control in the elderly within the framework of the shifting socioemotional motivations associated with older adults’ capacity for enhanced emotional processing, we hypothesize that a training program that simultaneously targets cognitive and emotional control and their neurobiological substrates would be more likely to successfully improve the overall quality of life in older adults. Mindfulness, as will be shown in this section, strengthens the neural circuitry associated with emotional and cognitive control. In addition, it has been shown to enhance the integrity of a critical resting-state system, the default-mode network, which underlies the functionality of these two inter-related networks. From this standpoint, the study of mindfulness appears to be uniquely capable of modifying not only the neuroarchitecture associated with controlled regulatory processing, but also the elementary infrastructure on which the brain’s baseline functioning is contingent (Figure 2).

### Mindfulness and emotion regulation

Emotion regulation, refers to the change in the subjective experience of emotion, including the temporal context in which it is experienced as well as the intensity and behavioral expression of that emotion (Gross, 1998). This individual capacity has important implications for decision-making, social functioning, psychopathological development, homeostatic processes, and overall quality of life.

Mindfulness, involving an attitudinal orientation of curiosity, openness, and acceptance, has been linked to emotional intelligence in its shared focus on perceptual clarity to one’s emotional state (Salovey and Mayer, 1990). A number of studies have in fact attributed the efficacy of mindfulness in reducing symptoms of stress and negative affect to its capacity to modify emotion regulation abilities (Arch and Craske, 2006; Chambers et al., 2008, 2009), with evidence suggesting that emotion regulation is directly engaged during the active performance of mindful exercises (Ortner et al., 2007; Farb et al., 2010). By enhancing behavioral self-regulation (Lykins and Baer, 2009), increasing emotional differentiation (Hill and Updegraff, 2012), and reducing the routine tendency to emotionally react to transitory thoughts and physical sensations (Teasdale et al., 2000; Ramel et al., 2004), mindfulness practice is thought to decrease negative affect, stress, and mood disturbance (Brown and Ryan, 2003), and protect against symptoms of anxiety and depression (Ostafin et al., 2006), including ruminative thinking (Jain et al., 2007; Deyo et al., 2009). In fact, core components of mindfulness have been integrated into clinical usage in therapeutic models ranging from Dialectical Behavioral Therapy (Linehan, 1994) to Mindfulness-Based Cognitive Therapy (Segal et al., 2002).

While there is unequivocal evidence for the role of mindfulness in enhancing overall emotional well-being and its associated facets, an interesting perspective, debating the involvement of bottom-up versus top-down neural control systems, has emerged in the neuroscientific study of mindfulness and emotional regulation (Chiesa et al., 2013). Much of this literature is in its relative infancy, and the heterogeneity of the adopted methods makes it slightly challenging to draw inferences and conclusions across studies. However, this diversity in methodology across various studies provides a platform upon which to evaluate the neural correlates of mindfulness in a number of different contexts. These include cross-sectional studies which examine it as a dispositional variable; short-term mindfulness training studies which aim to teach the skills of mindfulness to community-participants; as well as case studies of expert meditators, whose long-term practice of concentrative attention and open monitoring provide insights into the brain’s potential for neural plasticity following such intense mindful exercises. While comparing such varied studies can be fraught with its own set of challenges, including fluidity with the very definition of mindfulness (Grossman, 2011; Awasthi, 2013), such a comparison can also enable us to better understand the qualitative differences that might emerge based on the particular nature of meditative practice a given individual engages in. A critical question involves understanding what, if any, differential effect is observed when examining the impact of mindfulness on a novice practitioner who engages in this practice for 30–50 h of training, as is standard in a traditional mindfulness-based training program, versus an advanced practitioner who approaches mindfulness more as a value system and lifestyle, rather than a temporally-isolated experience in a training program.

Indeed, an examination of the association between mindfulness and the neural correlates of emotion regulation provides evidence for the recruitment of separate, yet inter-related processes of bottom-up and top-down emotional control in experts of mindfulness, relative to the short-term practitioners of mindfulness. Taylor et al. (2011) examined individual responses to emotional and neutral pictures from the International Affective Picture System (Lang et al., 1997) in a group of Zen meditators with experience of greater than 1000 h as compared to a group of novices. Participants were presented with these pictures during rest as well as during a condition that instructed them to engage in concentrative attention practice by making the breath as their anchor. Across participants, the mindfulness condition was associated with a decrease in emotional intensity, but the neural regions supporting such decreased emotional response were found to differ between experienced and novice practitioners. Specifically, novice practitioners were found to reduce activity in the bilateral amygdalae when processing negative or positive pictures, relative to the neutral stimuli, suggesting a down-regulation of this sub-cortical region during instructions of concentrative focus. Interestingly, the contrast of emotionally-intense stimuli with the neutral stimuli did not result in an up-regulation of the prefrontal cortices, as would be expected if processes of top-down control were activated. However, collapsing across valence categories indicated that novice, but not experienced meditators, showed an increased activation of the right dlPFC and the right posterior cingulate cortex (PCC) during the mindful condition. In here, the authors did not report a direct contrast between the novices versus the experienced meditators, so while these findings are preliminary and require replication, an intriguing possibility is that novice mediators might be engaging in emotional control through an up-regulation of the top-down control systems. Additionally, the study also reported that experienced meditators demonstrated a reduction in activity of the mPFC and the PCC across all valence categories, suggesting that a reduction in self-referential processing areas, rather than a reduced engagement of processes serving cognitive reappraisal, supports reduction in emotional reactivity in expert meditators. Further support for the engagement of the top-down circuitry during emotional experiences in controls is provided by studies of pain perception (Grant and Rainville, 2009; Grant et al., 2011; Gard et al., 2012). In such studies, control participants were able to reduce pain unpleasantness via the recruitment of the lateral prefrontal cortices, while expert meditators were found to successfully regulate the perception of pain through the recruitment of regions supporting viscero-somatic awareness, such as the posterior insula and the somatosensory cortex, and suppression of the activity of the dlPFC.

Complementary MRI research involving short-term mindfulness training programs (Allen et al., 2012) and studies of dispositional mindfulness as a trait variable (Creswell et al., 2007) also show evidence supporting the hypothesis that the engagement of cognitive reappraisal strategies underlies the reduction in emotional reactivity following a short-term training. In one such study, Creswell et al. (2007) examined the association between dispositional mindfulness and neural regions involved during an affect labeling task. They reported higher levels of dispositional mindfulness to be associated with increased recruitment of the dlPFC and reduced activity of the amygdala during labeling of affective stimuli, as compared to gender labeling. These results relating the construct of dispositional mindfulness with greater top-down involvement during the viewing of affective stimuli has been replicated in several other studies (Frewen et al., 2010; Modinos et al., 2010). These results provide support for the hypothesis that qualitative differences in the impact of mindfulness exist when this construct is treated as an idiosyncratic characteristic that varies in individuals ranging everywhere from community participants to expert meditators well-versed in practices of concentrative attention and open monitoring.

Similarly, short-term mindfulness-based studies also provide evidence for such engagement of the prefrontal cortices during affective regulation following limited training in principles and practices of mindfulness. In a recent study, Allen et al. (2012) examined alterations in neural recruitment during an emotional Stroop task in a group of participants that were randomized to a 6 week mindfulness training program, relative to participants in an active control group. Interestingly, post-intervention, participants in the mindfulness group showed enhanced recruitment of the dlPFC during executive processing, supporting the involvement of top-down control regions in short-term practitioners of mindfulness. Increased recruitment in the right anterior insula, ACC, as well as the mPFC during negative valence processing was observed only for individuals reporting greater engagement with the practices of mindfulness.

These studies collectively support a theoretical model of mindfulness and emotional regulation that offers the possibility of increased top-down regulation of the prefrontal cortices on the sub-cortical structures supporting emotional reactivity to increase emotional regulation in the early stages of mindfulness training. It seems likely that even though mindfulness training appears to be theoretically divergent from training that involves explicit cognitive appraisal, short-term training in this approach may in fact be associated with the engagement of systems supporting conscious cognitive regulation of affective experiences. With increased exercise of mindfulness skills and consistent engagement in consciously controlled emotion regulation, the practice of open monitoring, likely becomes habitual and pervasive. This shift, thereby enables an engagement of the neural circuitry supporting interoceptive awareness and somatosensory representation. For future research, it would be interesting to examine if a qualitative shift in the practice of mindfulness in novice participants is accompanied by a shift in reliance on the top-down regions to ones supporting sensory awareness.

### Mindfulness and cognitive control

At the crux of the construct of mindfulness lies its promotion of single-minded goal maintenance in the face of external or internal interference. The study of cognitive control, thus as an outcome variable has garnered critical interest in mindfulness-related research (Jha et al., 2007; Xiong and Doraiswamy, 2009; Chiesa et al., 2011; Malinowski, 2013). Both behavioral and neuroimaging studies, employing numerous variants of cognitive control tasks, have sought to determine whether training in mindfulness abilities, involving concentrative attention and open monitoring, are indeed associated with (1) an enhanced capacity to maintain a task-vigilant alert state, in which distractions arising from internal representations are prevented from interfering with task-set maintenance or task performance, and (2) better performance on tasks of executive control which require resolution of response competition through reduced interference from goal-irrelevant, external stimuli. As noted earlier, goal-neglect errors and related differences in working memory capacity during cognitive control performance have been suggested to stem in part from momentary failures of conscious thought control (Kane and Engle, 2003). Thus, mindfulness training might be theorized to directly relate to the controlled-attention component (i.e., the goal-maintenance component of the cognitive control of working memory), the successful functioning of which is thought to directly support executive control abilities and reduce response interference.

One of the widely used tasks in the mindfulness literature is the ANT, and as noted earlier, this task helps differentiate between three inter-related components or subsystems of attention: alerting, orienting, and executive control. The training of mindfulness skills has been found to enhance the executive control component of attention (Tang et al., 2007; Zeidan et al., 2010). This suggests that short-term training in skills of mindfulness can indeed enhance the ability to engage the top-down attentional control system and reduce distractor interference. Expert meditators, when compared to novices, have also been found to perform better on tasks tapping into response competition, such that sustained practices of focused attention result in higher levels of cognitive flexibility and executive control (Chan and Woollacott, 2007; Moore and Malinowski, 2009; van den Hurk et al., 2010a). Complementary neuroimaging evidence also provides support for the engagement of the PFC and the ACC during tasks of executive control, suggesting the further involvement of the neural circuitry sub-serving top-down cognitive control operations during the practice of mindfulness (Brefczynski-Lewis et al., 2007; Tang and Posner, 2009).

In addition to the malleability of the executive control component, research also provides support for the enhanced functioning of the alerting sub-system in expert meditators, relative to novices. The long-term cultivation of mindfulness, thus enhances the capacity to maintain a constant state of alertness, resulting in increased moment-to-moment awareness and thereby increasing the capacity for sustained attention (Lutz et al., 2008, 2009; Moore and Malinowski, 2009). In fact, extensive training with meditation practices was also found to counter the effects of an age-related decline in sustained attention in a group of Zen meditators (Pagnoni and Cekic, 2007), suggesting that such practices might be prophylactic for the decline in sustained attention or the alerting component of attention observed in the elderly (Gamboz et al., 2010).

Interestingly, mapping the emotional regulation literature, there appears to be some consistent differences surfacing between the techniques of expert practitioners, relative to individuals having undergone brief mindfulness-related programs. In the first direct test of these differences, Jha et al. (2007) compared performance on the ANT task between a group of meditation-naïve participants who engaged in a traditional 8 week MBSR program and a group of expert meditators who engaged in a 1 month intensive mindfulness retreat program, practicing the meditative skills for 10–12 h a day. While participation in the MBSR program enhanced the orienting skills of the participants, the expert meditators showed better performance on the alerting sub-component of the ANT task following the retreat, compared to participants in the MBSR group. Additionally, prior meditation experience was found to be correlated with the alerting sub-component, such that greater years of meditation practice was associated with better performance on this sub-component of attention. The results of this study provide first support for the hypothesis that a brief training program can engage the top-down attentional control system, thus improving aspects of voluntary attentional control. It also suggests that long-term engagement, resulting in the cultivation of a mindful state, is associated with the habitual promotion of sustained cognitive focus; possibly mediated through a reduction of distractions from goal-irrelevant representations and responses, arising both externally and internally.

Mirroring these behavioral findings, Brewer et al. (2011) found a differential engagement of the regions of the default-mode network in expert meditators, relative to novices, as they engaged in various meditation techniques. In this study, the authors examined the engagement of a critical network of the brain that has now been implicated in several functions, prominently including self-referential processing (Andrews-Hanna et al., 2007, 2010) and in mind-wandering during tasks of focused attention (Mason et al., 2007). The serendipitous discovery of the default-mode network as a task-negative network (Shulman et al., 1997; Raichle et al., 2001) that demonstrates deactivation during performance on tasks that require cognitive control operations has provided us with great insights into the functional architecture of the intrinsic networks of the brain and their coherence during exogenous and endogenous processing (Buckner et al., 2008; Biswal et al., 2010). The description of the default-mode network as the task-negative network of the brain stems primarily from its negative correlation with the CCN that is often engaged during tasks of response competition (Fox et al., 2005; Hagmann et al., 2008; Buckner et al., 2009; Cole et al., 2010). The activation of regions of the default-mode network during studies of attentional control has been found to be associated with poor performance (Shulman et al., 2007; Anticevic et al., 2010), mind-wandering (Mason et al., 2007; Christoff et al., 2009), task-irrelevant thoughts (McKiernan et al., 2003, 2006; Stawarczyk et al., 2011), and maladaptive ruminative thinking (Hamilton et al., 2011). In fact, recently Smallwood et al. (2013) found that mind wandering, indexed by trials preceding errors on a task of cognitive control, was associated with activation of the regions of the default-mode network. Given that neural activity prior to committing an error likely represents a lapse in attentional control, the activation of the regions comprising the default-mode network suggest the engagement of these regions when attention is diverted from the current context to inward processing.

Corroborating this involvement of the default-mode network regions, specifically the mPFC, as well as the PCC during mind-wandering, Brewer et al. (2011) found support for the suppression of neural activity in these regions in expert meditators when engaged in different meditation practices, relative to demographically-matched controls. That is, by continuously exercising the selective direction of attention, long-term practitioners of mindfulness were able to appropriately modulate task-relevant and irrelevant regions, thereby strengthening the integrity of the various intrinsic networks. The engagement of the default-mode network during mind-wandering in meditators was further substantiated in another recent study by Hasenkamp et al. (2012) which examined the neural correlates of four stages of a cognitive cycle. These included, the presence of mind-wandering, the awareness that mind-wandering has indeed taken place, the shifting of attention to the present moment, and finally the maintenance of attention. Mind-wandering in expert meditators, was found to engage the PCC, the mPFC, the posterior parietal/temporal cortex, and the parahippocampal gyrus, all nodes of the defaut-mode network (DMN). Sustained attention and the shifting of attention were, in fact, associated with the activity of the dlPFC and the parietal cortices, thus, corroborating previous research on the fronto-parietal network and its involvement in sustaining attention.

While still preliminary and needing replication, these studies suggest that brief mindfulness training programs that cultivate both focused attention and open monitoring allow the development of enhanced cognitive control operations. This is thought to take place primarily through the engagement of the top-down cognitive control system, with the frontal and parietal cortices being critically involved in the selection of task-relevant attributes and suppression of task-irrelevant attributes (Figure 2). Long-term engagement in the practices of focused attention, and subsequent open monitoring via receptive attention to the plethora of events unfolding on a moment-to-moment basis creates a state of preparedness and alertness by fundamentally reducing vulnerability to the everyday reactivity and distractions of the wandering mind (Bishop et al., 2004). The result is a more flexible modulation of neural networks supporting various stages of an attentional state and the subsequent strengthening of these critical networks. Taken together, the training of mindfulness skills appears to have the potential to influence the inter-related circuitry of the cognitive control network, as well as the default-mode network. Given the deficits seen in the recruitment and suppression of both these networks in older adults per the demands of the task (Prakash et al., 2009, 2012), mindfulness training may be a viable solution for the deficits in cognitive control seen in older adults. By reducing the distractions arising from external stimuli, as well as internal disruptions, long-term training with mindfulness can, in fact, result in a task-dependent modulation of the critical networks of the brain, which may enhance cognitive performance and likely reduce rumination.

## Future directions and key considerations

**Figure 3:**
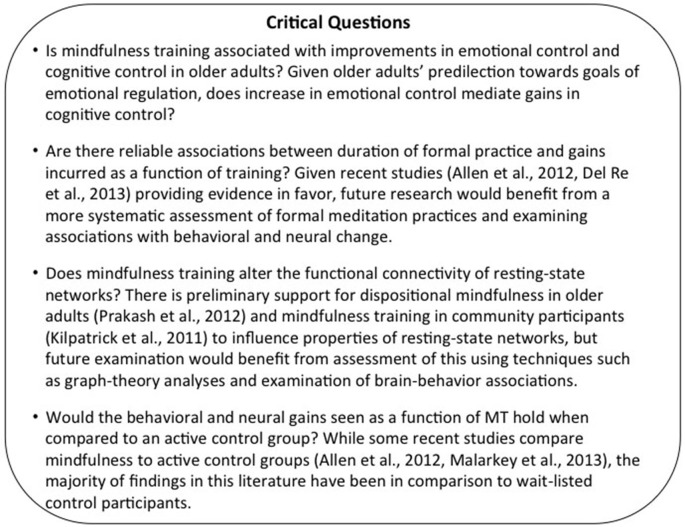
**Highlights the key points from the section on Future Directions and Key Considerations**.

The above-reviewed literature provides support for the potential of mindfulness training in improving controlled regulatory processing. By virtue of enhancing one-minded concentration and sustained attention, mindfulness training gradually cultivates the development of attentional skills that promote goal maintenance, while reducing distractions not just from external, competing stimuli, but also from the internal disruptions and wanderings of the mind (Mrazek et al., 2012, 2013). In so doing, mindfulness qualitatively enhances our ability to engage in more conscious top-down cognitive control through the recruitment of the lPFC, gradually promoting a state of vigilance and sustained attention that may be extended to all objects of attention. The result of years of such concentrative attention seems to be a shift from reliance on the prefrontal cortices, known to be critically involved in consciously controlled top-down attentional control, to a more bottom-up recruitment of the viscero-somatic regions supporting a flexible modulation of the intrinsic networks of the human brain (van den Hurk et al., 2010b; Josipovic et al., 2012). Extending this to the domain of emotional regulation, mindfulness training has the potential to enhance overall well-being, primarily by engaging conscious awareness at every level of each action and perception in the present moments, eventually resulting in a reduced tendency to behave reflexively. Teaching individuals to cultivate an openness to the experiences of the present moment, by taking the stance of an impartial witness, mindfulness training specifically promotes the development of emotion regulation rather than emotional reactivity. Older adults, with their predilection to engage in effortful emotional regulation, are thus, likely to benefit from a training program that purports to further enhance and strengthen their skills of emotional regulation, and in the process of doing so, also improve the facets of cognitive control that decline with aging. Thus, a critical direction for future research is to examine if mindfulness training is in fact associated with increases in emotional regulation and cognitive control in older adults (see Figure 3 for key directions for future research). Evidence for the efficacy of this low-cost intervention in incurring broad gains in cognitive and emotional functioning would contribute significantly to the field of cognitive aging.

This area of research has further been strengthened by the growing contributions of neuroimaging studies aiming to examine the contributions of cortical and sub-cortical regions in cognitive and emotional control. The involvement of the lPFC in both emotional and cognitive control suggests the possibility of neural overlap in these two inter-dependent processes. This, in turn, has the potential to predict functional overlap, which is associated with broad transfer effects (Lustig et al., 2009). Previous studies of cognitive training have, in fact, provided evidence for the possibility of broad transfer effects to functional tasks when neural overlap was present between the trained task and the transfer task. In such a study, Dahlin et al. (2008) found evidence for transfer of training in young adults, and not older adults, primarily due to the absence of shared neural recruitment between the trained task (memory updating) and the transfer task (working memory) in older adults. The authors concluded that it was the reliance on a shared neural circuitry that was critical for the presence of broad transfer effects. Given the shared circuitry of emotional and cognitive control in older adults, mindfulness training, by virtue of enhancing emotional control, also has the potential to increase cognitive performance. Of course, an interesting line of future research would be the examination of whether it is the increase in emotion regulation capabilities that fosters greater cognitive control or the development of cognitive control capacity that is a necessary pre-requisite for increased emotional regulation? Research has just started to answer this question of a temporal evolution of cognitive and emotional control capacities in the context of mindfulness practices (Sahdra et al., 2011), with studies providing preliminary evidence supporting cognitive control capacities as precursors to the development of enhanced emotional regulation. However, it would be important to examine the relative change in emotional and cognitive control capacities in an aging population that places greater emphasis on emotional regulation skills. It would be interesting in future research to systematically examine the evolution of enhanced cognitive and emotional capacity in both an older and young cohort in order to systematically differentiate the effect of mindfulness training on these inter-related capacities as a function of age.

Another critical theme that emerges from the above reviewed literature is the presence of qualitative differences in neural engagement in long-term practitioners of mindfulness, relative to short-term practitioners of the techniques. Transliterating these findings to the study of mindfulness, it is critical for future studies to systematically examine the amount of time spent by participants engaging in the formal practice of mindfulness during the 8 week period. Short-term training studies that have investigated the association between time spent in formal practice and psychological and neural benefits evince support for the critical variance explained by engaging in such formal practices of concentrative attention and open monitoring (Allen et al., 2012; Del Re et al., 2013). While novice meditators tend to recruit more of the top-down circuitry of the lateral prefrontal cortices, expert meditators and those engaging in greater amounts of practice in an 8 week program show evidence for the involvement of the viscero-somatic regions of the somato-sensory cortices, insular cortices, and regions of the default-mode network.

Interestingly, in our own research, we have found evidence for an association between higher levels of dispositional mindfulness and connectivity of the default-mode network in older adults (Prakash et al., 2013). In this study, participants completed a self-report measure of mindfulness disposition (Mindful Attention and Awareness Scale, MAAS; Brown and Ryan, 2003) and completed a resting-state scan. Network integrity was examined in the default-mode network, given recent investigations of the involvement of this network in self-referential processing and its associated negative correlations with the cognitive control network. Higher levels of mindfulness disposition in older adults were found to be associated with connectivity of the dorsal PCC, such that the increased connectivity of this area with the rest of the default-mode network provided evidence for greater neuronal integrity of this area with the DMN. Interestingly, the dorsal PCC has been implicated more as a switch area that works at the interface of the cognitive control and default-mode networks (Leech et al., 2011). The involvement of this area, relative to the ventral PCC which is often implicated in the traditional default-mode network, provides evidence suggesting that mindfulness disposition may be associated with the flexible modulation of neuronal regions. Future work would need to examine the extent to which the findings of a cross-sectional study mirror the results of a short-term training program, as recent conceptualizations of mindfulness would suggest qualitative differences between examining this construct as a trait measure versus examining it as an acquired skill honed through the practice of concentrative attention and a non-judgmental attitude.

Finally, keeping in perspective the growing interest in intervention studies that “train the brain”, it is critical to examine the efficacy of mindfulness training, relative to an active control group, that reduces participant bias and accounts for the amount of time spent by participants engaging in activities related to the program. While these threats to internal validity of a randomized controlled trial have been well-known for a while (Campbell and Stanley, 1963; Shadish et al., 2002), much of the training literature (and not just the mindfulness literature) tends to ignore the possible influence of participant motivation on task performance (see Boot et al., 2011; Boot and Simons, 2012 for a critical discussion of this issue). While it is our position that wait-listed studies provide a critical cost-effective, pragmatic approach to generating hypotheses about the outcome and the mechanisms underlying a training approach, investigative efforts moving forward need to examine the effects of mindfulness interventions, relative to more active control programs.

The unique capacity for flexible, consciously-controlled, dynamic behavior distinguishes the human species from all others, providing us with the seeds of adaptability and creativity and granting us a communicable sense of free will and a degree of control over the environment. In the current work, we present a comprehensive review of how cognitive and emotional control change, behaviorally and neurally, as a function of age and examine how a mindfulness-based model may have the potential to counteract such nuanced age-related alterations in controlled regulatory processing. It is hoped that the comprehensive review provided in this paper may be somehow used to guide future investigations of emotion-cognition processes in the elderly. By developing more refined methods of examining age-related changes in cognitive, affective, and neural functioning, we move closer towards understanding the complex metamorphosis of the aging brain and our powerful role in relationship to it.

## Conflict of interest statement

The authors declare that the research was conducted in the absence of any commercial or financial relationships that could be construed as a potential conflict of interest.
